# Quality of Life in Individuals with Intellectual and Developmental Disabilities: The Congruency Effect between Reports

**DOI:** 10.3390/healthcare11121748

**Published:** 2023-06-14

**Authors:** Miguel Jacinto, Filipe Rodrigues, Diogo Monteiro, Raul Antunes, José Pedro Ferreira, Rui Matos, Maria João Campos

**Affiliations:** 1 Faculty of Sport Sciences and Physical Education, University of Coimbra, 3040-248 Coimbra, Portugal; migueljacinto1995@gmail.com (M.J.); jpferreira@fcdef.uc.pt (J.P.F.); mjcampos@fcdef.uc.pt (M.J.C.); 2 ESECS, Polytechnic of Leiria, 2411-901 Leiria, Portugal; filipe.rodrigues@ipleiria.pt (F.R.); diogo.monteiro@ipleiria.pt (D.M.); rui.matos@ipleiria.pt (R.M.); 3 Life Quality Research Centre (CIEQV), 2040-413 Rio Maior, Portugal; 4 Research Center in Sport Sciences, Health Sciences and Human Development (CIDESD), 5001-801 Vila Real, Portugal; 5 Center for Innovative Care and Health Technology (ciTechCare), Polytechnic of Leiria, 2411-901 Leiria, Portugal; 6 Research Center for Sport and Physical Activity (CIDAF), 3040-248 Coimbra, Portugal

**Keywords:** caregivers/family members, intellectual disability, *Escala Pessoal de Resultados*, Personal Outcomes Scale, self-reports

## Abstract

Assessing quality of life (QoL) is important to provide personalized and individualized support plans with the purpose of improving personal outcomes. Based on the conceptual model of QoL, the aim of this study was to assess the congruence between the perceptions of institutionalized individuals with intellectual and developmental disabilities (IDD) and the perception of a third party, regarding QoL. Forty-two individuals participated in this study, including twenty-one with mild to severe IDD and their family members/caregiver/reference technician, who responded to the Personal Outcomes Scale (Portuguese version). Significant differences (*p* < 0.05) were found between reports in the personal development (*t* = −2.26; *p* = 0.024), emotional well-being (*t* = −2.263; *p* = 0.024), physical well-being (*t* = −2.491; *p* = 0.013) and total QoL (*t* = −2.331; *p* = 0.02). The results further show that most third-party reports tend to undervalue the QoL of the individual with IDD, and that there is no congruence in any of the QoL domains. The inclusion of self-reports in the QoL assessment is important. In addition to the assessment of third-party reports, the process of making decisions appropriate to the context and individual characteristics is equally important. On the other hand, the inclusion of third-party reports is an opportunity to promote communication among all stakeholders, recognize and discuss differences, and promote QoL, not only of individuals with IDD, but also of families.

## 1. Introduction

Quality of life (QoL), for Barbosa [1], is expressed by the relationship between male, nature and the environment that surrounds him, forming a whole.

Minayo et al. [2] states that QoL is a personal concept, related to the degree of satisfaction in family, love, social, environmental, and existential aesthetics. For this author, the QoL concept expresses knowledge, individual values, and experiences, as well as the associations and cultures belonging to certain moments, spaces, times, and histories.

For Vilarta [3] QoL is the way in which subjects live, feel and understand their daily lives, encompassing aspects such as housing, health, education, transport, work and participation in decision-making about them. This notion delimits the conditions in which human beings live, resulting from a set of individual, environmental and social factors [4]. It can also be defined as the distance between individual expectation and reality, given that the greater the distance, the worse the QoL [5].

On the other hand, for the World Health Organization [6,7], QoL concerns the individual’s perceptions of their position in life, in the context, culture and value system to which they are inserted, taking into account the relationship with their goals, expectations, standards and concerns. The WHOQOL is a multidimensional instrument that has been validated assuming seven domains, namely: (i) physical health; (ii) psychological domain; (iii) level of independence; (iv) social relationships; (v) environment; (vi) spirituality; and (vii) personal domain.

There are different definitions of QoL (as we have shown above) and, although they diverge in the construct, they converge as to the existence of multidimensional domains and respective indicators [8]. For this study, we considered the construct presented by Schalock et al. [9], because this concept is one of the most cited, used, and has critical impact on research or in practice, involving individuals with the characteristics of the participants in this study. Schalock et al. [9] refers to QoL as a set of factors that address individual well-being or the perception of their social position, in the context and culture to which they are inserted, assuming sociocultural values, needs, expectations and individual preferences. It is a multidimensional phenomenon composed of factors and domains (Table 1), influenced by personal characteristics and environmental contexts [10].

The independence factor comprises the domains of “Personal Development” and “Self-determination”, which reflect the degree of autonomy of the individual. The domains of “Interpersonal Relations”, “Social Inclusion” and “Rights” reflect the Social Participation factor. Lastly, the domains of “Emotional Well-Being”, “Physical Well-Being” and “Material Well-Being” correspond to the Well-Being factor. The domain of “Personal Development” is related to individual lifelong learning, including education, as well as the ability to acquire skills and/or abilities, and to demonstrate them in the community. The domain of “Self-Determination” relates to personal objectives, goals, and desires, and the ability to make decisions and to make one’s own choices. The domain of “Interpersonal Relationships” is related to relationships with others, with respect to family, friends, and/or social networks. It is also related to the support and help you receive from others. Regarding the “Social Inclusion” domain, as the name implies, it is related to inclusion and participation in the community, as well as the roles one plays in it and also the support one receives from society. Contained in the “Rights” domain, are aspects such as respect, dignity, equality, citizenship, access, and fair treatment. The “Emotional Well-Being” domain is related to perceived life satisfaction, self-concept, and absence of stress. The “Physical Well-Being” domain is related to overall health, namely health care, ability to take care of oneself, mobility, and recreation/leisure. Ending with the domain of “Material Well-Being”, which contains questions that address financial status, employment, as well as living conditions and material possessions that one has [11].

The concept and study of QoL in individuals with Intellectual Developmental Disabilities—IDD (i.e., Intellectual Disability) has been gaining interest from various stakeholders, having practical implications for interventions that are performed for and with this population [9], in order to drive progress towards equity, empowerment, and self-determination [12]. In individuals with IDD, characterized by a deficit of intellectual and adaptive functioning in the conceptual, social and practical domains, identified with mild, moderate, severe and profound degrees and developed before 22 years old [13], measuring QoL allows: (i) to understand their degree of satisfaction; (ii) understand personal perceptions; (iii) support decision-making; (iv) evaluate the intervention; and (v) evaluate theoretical models. This measurement allows researchers to direct the individual to the life they like and their values [14]. On the other hand, measuring QoL in individuals with IDD aims to address challenges and overcome barriers that people with IDD have been facing, as well as to improve public policies or service practices to meet their needs and choices.

QoL scales for people with IDD, and each indicator should be assessed by two methods: subjectively, involving the person, they/themself, as a primary respondent (self-reports); and objectively, based on proxy reports of the person’s experiences and circumstances. Self-report measures are widely accepted for assessing QoL. However, it is also important to consider the views of people who know a person well (measures of others’ reports). Self-report measures for people with IDD have become essential in this field of research because they actively participate in taking on their role as individual citizens [15]. On the other hand, the reporting of others allows us to observe potential differences between people with IDD and support staff or family members [16,17]. The combination of the two measures provides an estimate of the accuracy of people with IDD reporting, enriching information for informative decision-making, which is an important step in increasing the ongoing understanding of how to assess and improve the QoL of people with IDD.

In the present investigation, we aimed to align these trends and discuss the relationship between self-report and proxy-report measures. The aim of the study was to explore different perceptions in QoL assessments by examining the effect of congruency between the perceived QoL of 21 individuals with IDD, institutionalized in a supportive care organization, and the 21 family members/caregiver/reference technician perceptions. For the present study, the following hypotheses were defined: (i) self-reports have higher QoL values; (ii) third-party reports have higher values; and (iii) there are no significant differences between reports. Knowing that individuals with IDD demonstrate low levels of QoL, and therefore, their relatives and/or reference technician (caregiver) [18] are impacted, analyzing the different responses is an opportunity to promote communication between all stakeholders, recognize and discuss differences, and promote QoL, not only for individuals with IDD, but also for families.

## 2. Materials and Methods

This cross-sectional study was carried out in accordance with the Declaration of Helsinki for human studies [19] and was approved by the ethics committee of the University of Coimbra—Faculty of Sport Sciences and Physical Education with the approval code: CE/FCDEF-UC/00872021.

### 2.1. Participants

Twenty-one individuals volunteered (42.81 ± 10.99 years; 10 females and 11 males), who were institutionalized in a support institution, located in Leiria, Portugal, recruited by the non-probabilistic convenience method. Their family members/caregiver/reference technician also participated in the study. The following inclusion criteria were defined as: (1) aged ≥18 years; (2) adults with mild, moderate, or severe IDD and their family members/caregiver/reference technician; and (3) capacity to individually complete the questionnaires. Exclusion criteria were defined as: (1) individuals with other associated pathologies; (2) profound IDD; (3) inability to communicate; and (4) non-delivery of signed informed consent.

### 2.2. Quality of Life Assessment/Instrument

For a correct assessment of personal outcomes, it is necessary to have measures with satisfactory psychometric properties, based on an empirically validated model. As stated in the principles of QoL measurement, the assessment involves the combination of the subjective measure of well-being (including individual preferences) and the objective circumstances and life experiences. The Personal Outcomes Scale [10,20,21,22] is a measure developed according to several studies encompassing the conceptualization and validation of the different QoL domains. This measure allows to: (i) understand their degree of individual satisfaction; (ii) understand personal perceptions; (iii) support decision-making; (iv) evaluate the intervention; and (v) evaluate theoretical models. This measure allows us to direct the individual to the life they like and their values, and is designed to assess, firstly, people with IDD (self-reports) and, secondly, the perspectives of people close to them (professional or family—third-party report) [14].

The Personal Outcomes Scale (Portuguese version) [15] was applied by technicians with specific training for this purpose. As in the original version [20,22], the scale has two parts: (a) questions to be answered by the individual with IDD (self-report); and (b) questions to be answered by a family members/caregiver/reference technician (third-party report). The scale is composed of a total of 48 questions in each part (6 questions per domain). Each item is rated on a 3-point Likert scale, and a higher score indicates better QoL (e.g., 3 = always; 2 = sometimes; 1 = seldom or never).

For self-report measure, all composite reliability coefficients were within standards for acceptable internal consistency ranging from 0.75 to 0.91. For third-party report measure, the scores ranged from 0.72 to 0.92.

### 2.3. Procedure

The same procedures of a previous study [23] was applied, which should be consulted for a more detailed knowledge of the procedure used in the present study. All assessments were performed in the support institution by the same researchers to minimize possible measurement errors. Instructions were provided to ensure the safety and comfort of the participants. The application of the scale will be carried out in a room with isolation from noise and possible distractions, in a 1:1 aspect (one specialist for one participant) for individuals with IDD and their family members/caregiver/reference technician. For each individual with IDD, we collected two responses: (i) self-reports and (ii) third-party reports.

### 2.4. Statistical Analysis

Descriptive statistics were used to characterize the sample, including mean, standard deviation, median, and minimum and maximum. The *Shapiro–Wilk* and *Levene* test were used to test the normality of the results. The level of agreement, under-reporting, and over-reporting between the ratings of the individuals with IDD themselves and their family members/caregiver/reference technician were calculated to see if they agreed or disagreed. The minimum level was set at 10% discrepancy, as suggested in previous studies [24,25]. Then, the IDD individuals’ own scores were subtracted from the scores of their family members/caregiver/reference technician, since individual perceptions may be the most meaningful measure [26]. The following calculations were then performed: (i) the percentage of agreeing behaviors, defined as less than half the standard deviation (−0.5/0.5) between both scores; (ii) the percentage of over-reported behaviors, explained by the mean scores of family members/caregiver/reference technician who were 0.5 standard deviation above the self-perception of individuals with IDD; and (iii) the percentage of under-reported QoL, defined as the mean scores which are below half the standard deviation. The middle deviation criterion was based on previous assumptions [27] and the cut-offs are considered as a reliable source of group characterization. In the final step, the mean scores for all domains of the interpersonal QoL were compared with the categorization of the three groups. The existence of significant differences between groups will be analyzed using the *Wilcoxon* test. All data were analyzed using IBM SPSS Statistics (version 28, IBM Corporation (SPSS Inc., Chicago, IL, USA) and the significance level adopted was *p* < 0.05.

## 3. Results

The mean rates of discrepancies and agreement are presented in Table 2. The results seem support a rather unbalanced distribution, where disagreement seems to exist in all QoL domains, with participants perceiving higher values compared to those reported by family members/caregiver/reference technician. After performing the statistical test that compares the reports, the differences between responses are only significant for the personal development (*t* = −2.26; *p* = 0.024), emotional well-being (*t* = −2.263; *p* = 0.02), physical well-being (*t* = −2.491; *p* = 0.01) and total QoL (*t* = −2.331; *p* = 0.02).

## 4. Discussion

The objective of this study was to compare the agreement and discrepancies between self-report and third-party answers on the perceived QoL of individuals with IDD.

In cases showing disagreement regarding the level of perception of QoL, the results supported that individual with IDD perceived higher values than their family members/caregiver/reference technician. Then, we can confirm hypothesis one. This fact seemed to be true for all domains of the QoL scale, however, was only significant for “Personal development”, “Emotional well-being”, “Physical well-being” and total QoL. According to the Conceptual Model of QoL [10], the sample in this study appears to perceive higher values of “Personal development”, including education, as well as the ability to acquire skills and/or abilities and show them in the community, and shows that the IDD individuals have a higher perception in their developmental abilities than their family members/caregiver/reference technician. Similarly, individuals with IDD perceive themselves as having more abilities compared to self-reports for the domain “Emotional well-being” (perceived life satisfaction, self-concept, and lack of stress) and the domain “Physical well-being” (general health, including health care, ability to care for oneself, mobility, and recreation/leisure).

The literature evidences a high agreement between self-reports and other reports, highlighting the fact that the information complements each other [20,28]; results that were not found in our study. The severity of the disability may predict a higher disagreement between answers [29], or the IQ level with the discrepancy in some domains [30], which may be the justification for our results. On the other hand, there is evidence that the degree of agreement may depend on whether the reporter is a family member or a professional, and professionals’ reports were closer to self-reports than to family reports [31]. This level of agreement seems to depend more on the degree of closeness and relationship in everyday life than the professional nature or family relationship [28,29]. On the other hand, caring is also associated with a burden and can be stressful and can have an effect on the caregiver’s own QoL [32,33], which can, in turn, influence the perception of the individual with IDD. Our study is in agreement with other studies, in other contexts/populations, where the answers of family members/caregiver/reference technician tend to underestimate the QoL scores, which may be associated with a less concrete and visible perception of certain domains of QoL, or that the level of agreement decreases as the disability/illness becomes more severe [34,35,36,37]. On the other hand, according to Milne et al. [37], these discrepancies may not be justified by a lack of agreement, but rather by methodological weaknesses, such as differences in interpretations of response categories, and may be influenced by other factors, such as caregiver anxieties and patient stoicism.

This discrepancy between reports cannot be seen as a question of unreliability and a discussion about who is “right” or “wrong”, but rather an opportunity to combine both perceptions for a more correct adjustment and response to the needs of all stakeholders [38]. In addition, it is an opportunity to promote communication between all stakeholders, recognize and discuss differences, and promote the QoL, not only of individuals with IDD, but also of families.

Although we could not examine more specifically whether the results could be replicated for different peers due to sampling limitations, our results are consistent with some of the previous studies [39,40]. These results further highlight the importance of self-report measures of QoL, and the integration of proxy reports to provide more importance to and, go beyond one-way perspectives, by considering both self-report and family member/reference technician perspectives, as these responses do not always agree.

However, family members do not always undervalue the QoL of individuals with IDD. In the study by Berástegui et al. [41], individuals with IDD scored lower in the domains (physical well-being, material well-being, and rights). On the other hand, individuals with IDD perceived higher levels of QoL in the Emotional Well-being and Physical well-being domains, in agreement with previous studies [42], indicating that support strategies should focus on maintaining these values and increasing the remaining domains.

Despite some studies showing the existence of barriers in self-reports, namely, the difficulty that some people with disabilities have in understanding the questionnaire or communicating their perspectives, needs and feelings, and the difficulty of data collection, namely, the collection of informed consent and the bias that the response may have [43,44,45,46], there is a growing interest and commitment to consider the report of individuals with IDD in the assessment of their QoL [20,43,44]. For all domains of QoL, responses include an interaction between objective circumstances and subjective perceptions, evaluations, and feelings about them, and self-reports play an essential role in accurately assessing their QoL. An assessment of QoL without considering self-reports translates into a contradiction, in the sense that decision-making is focused on Person-centered planning [47].

Considering that there are 200 million individuals with IDD worldwide (more or less 2.6% of the world population) [48], the fact that individuals with IDD have lower QoL values compared to non-disabled individuals [42,49] and that QoL decreases with advancing disability and age [50], a continued commitment to specialized support, assistance, and services is needed. On the other hand, the growing increase of individuals with IDD in support institutions makes the study of QoL relevant. According to the United Nations Convention, human rights are equal for all and exercising them is a responsibility of society. It is necessary to change rules and regulations, but also to transform society, starting by transforming support organizations, empowering people with IDD and their families, improving professional and organizational practices, and taking a qualitative leap in the strategic planning of public policies, in order to improve the QoL of individuals with IDD. [51].

The cross-sectional nature of the study does not allow inferences about the degree of fluctuation of the results over time. At the same time, due to the small number of participants in this study and the convenience sampling, the results should be considered with caution. On the other hand, the QoL of people with IDD is significantly affected by micro-, meso-, and macro-factors, and should be the focus of future research. According to Bronfenbrenner’s Ecological Systems model [52], each of these systems contains functions, norms, and rules that can influence an individual’s development, which must be seen as an integral part of the process. Individual, organizational, and systemic factors simultaneously impact experience and livelihood of people with IDD; institutionalization and the timing of their survey may have affected the results [53].

To obtain a more complete picture of the QoL of individuals with IDD, further research is needed to determine the effects of various factors on QoL, monitor the success of the interventions applied, and determine effective and adapted guidelines/strategies in the intervention with this population. These strategies for improving QoL among the population with IDD should be transversal to all institutions and their respective professionals, as well as to family members, with the aim of improving personal outcomes. Future studies should further explore the relationship between functionality/physical fitness and QoL [54,55].

## 5. Conclusions

Assessing the QoL of individuals with IDD is essential to detect, monitor, and report their support needs, and to carry out effective and adapted individual, institutional, and policy planning.

Our sample with IDD seems to perceive higher values for all domains of QoL. However, the significant differences between responses are only significant for the personal development, emotional well-being, physical well-being and total QoL.

In addition, the results of this study deepen the understanding of the complexity of the QoL assessment process of individuals with IDD. They highlight that it is important not only to measure the perceptions of QoL of individuals with IDD through the perceptions of their family members, but also through self-reports; both accounts being complementary could negatively impacxt QoL of people with ID, if their self-report views are not respectfully considered when planning strategies.

## Figures and Tables

**Table 1:** This is a Conceptual Model of QoL.

Factor	Domain	Indicators
Independence	Personal development	Activities of daily living, adaptive behaviour
	Self-determination	Personal choices, decisions and objectives
Social Participation	Interpersonal relations	Social activities and friendships
	Social inclusion	Social inclusion/community involvement
	Rights	Human and legal
Well-being	Emotional	Protection and security and absence of stress
	Physical	Health, nutrition, sport, recreation and leisure (variables that talk about physical variables)
	Material	Employment and economic status

Note: Source: Schalock et al., [10].

**Table 2:** Frequencies of predictor discrepancies and agreement.

QoL Domains	Third Party Report			Self-Report			Congruence Effects		
	Mean ± SD	Median	Min-Max	Mean ± SD	Median	Min-Max	Differences between Reports	%	Comparison between Reports
Personal development	8.71 ± 1.45	8.85	6–11	9.57 ± 1.91	9.78	7–14	Under-report answers family members/caregiver/reference technician > self-reports	14.3	*t* = −2.26; *p* = 0.02
							Agreement = answers family members/caregiver/reference technician = self-reports	23.8	
							Over-report answers family members/caregiver/reference technician < self-reports	61.9	
Self-determination	10.14 ± 2.55	10.57	5–15	11.14 ± 2.10	11.07	7–15	Under-report answers family members/caregiver/reference technician > self-reports	23.8	*t* = −1.667; *p* > 0.05
							Agreement = answers family members/caregiver/reference technician = self-reports	19	
							Over-report answers family members/caregiver/reference technician < self-reports	57.1	
Interpersonal relations	10.43 ± 1.85	9.71	8–15	11.38 ± 2.2	9.19	7–15	Under-report answers family members/caregiver/reference technician > self-reports	28.6	*t* = −1.749; *p* > 0.05
							Agreement = answers family members/caregiver/reference technician = self-reports	23.8	
							Over-report answers family members/caregiver/reference technician < self-reports	47.6	
Social inclusion	9.71 ± 1.97	8.85	6–13	10.71 ± 2.39	9.35	6–15	Under-report answers family members/caregiver/reference technician > self-reports	28.6	*t* = −1.094; *p* > 0.05
							Agreement = answers family members/caregiver/reference technician = self-reports	14.3	
							Over-report answers family members/caregiver/reference technician < self-reports	57.1	
Rights	9 ± 2.23	10	5–14	9.43 ± 1.77	9.71	6–13	Under-report answers family members/caregiver/reference technician > self-reports	33.3	*t* = −0.962; *p* > 0.05
							Agreement = answers caregiver/reference technician = self-reports	28.6	
							Over-report answers family members/caregiver/reference technician < self-reports	38.1	
Emotional Well-being	12.38 ± 1.65	12.19	8–14	13.62 ± 1.56	11.8	10–15	Under-report answers family members/caregiver/reference technician > self-reports	23.8	*t* = −2.263; *p* = 0.02
							Agreement = answers family members/caregiver/reference technician = self-reports	14.3	
							Over-report answers family members/caregiver/reference technician < self-reports	61.9	
Physical Well-being	11.95 ± 2.01	12.47	7–15	13 ± 1.44	12.5	10–15	Under-report answers family members/caregiver/reference technician > self-reports	19	*t* = −2.491; *p* = 0.01
							Agreement = answers family members/caregiver/reference technician = self-reports	28.6	
							Over-report answers family members/caregiver/reference technician < self-reports	52.4	
Material Well-being	7.48 ± 1.53	6.73	5–10	7.76 ± 2.04	2.04	5–12	Under-report answers family members/caregiver/reference technician > self-reports	23.8	*t* = −1.667; *p* > 0.05
							Agreement = answers family members/caregiver/reference technician = self-reports	28.6	
							Over-report answers family members/caregiver/reference technician < self-reports	47.6	
Total QoL	79.81 ± 8.04	79.4	63–91	86.62 ± 8.31	7.38	71–101	Under-report answers family members/caregiver/reference technician > self-reports	33.3	*t* = −2.331; *p* = 0.02
							Agreement = answers family members/caregiver/reference technician = self-reports	0	
							Over-report answers family members/caregiver/reference technician < self-reports	66.7	

QoL, quality of life; *p*, *p* value between reports; *t*, test value; SD, standard deviation; %, percentage.

## Data Availability

The data presented in this study are available on request from the corresponding author.
